# Evaluating clinical and laboratory effects of ozone in non-surgical periodontal treatment: a randomized controlled trial

**DOI:** 10.1590/1678-7757-2018-0108

**Published:** 2019-01-14

**Authors:** Eltas Seydanur Dengizek, Dundar Serkan, Eltas Abubekir, Karabulut Aysun Bay, Otlu Onder, Cicek Arife

**Affiliations:** 1 Canakkale Onsekiz Mart University, Faculty of Dentistry, Department of Periodontology, Canakkale, Turkey; 2 Firat University, Faculty of Dentistry, Department of Periodontology, Elazıg, Turkey; 3 Yildirim Beyazit University, Faculty of Medicine, Department of Biochemistry, Ankara, Turkey; 4 Firat University, Faculty of Veterinary, Elazıg, Turkey; 5 İnonu University, Faculty of Dentistry, Department of Periodontology, Malatya, Turkey

**Keywords:** Chronic periodontitis, Inflammation, Ozone, Periodontal treatment, TGF-β

## Abstract

**Objective:**

This study aims to evaluate the clinical and biochemical (oxidative stress and pro-inflammatory mediators) effects of the gaseous ozone use accompanied by scaling and root planning (SRP) in periodontal treatment.

**Material and Methods:**

The study population consisted of 40 patients with chronic periodontitis (CP) randomly sorted into two groups of 20. The experimental group received SRP plus 3 watts gaseous ozone in two separate applications five days apart, whereas the control group received SRP plus placebo. Clinical periodontal parameters were assayed and saliva samples were taken before the initial and one month after the second treatment. Periodontal examination assessed plaque index (PI), gingival index (GI), probing depth, and clinical attachment level (CAL). Total antioxidant status (TAS), total oxidant status (TOS), nitric oxide (NO), 8-hydroxy-2'-deoxyguanosine (8-OHdG), myeloperoxidase (MPO), glutathione (GSH), malondialdehyde (MDA), and transforming growth factor-beta (TGF-β) levels were evaluated from saliva samples.

**Results:**

Changes following treatment in PI, GI, probing depth, and CAL scores were similar for both groups (p>0.05). Of note, TGF-β levels were observed to be higher in the treatment group than in controls (p<0.05). Changes in 8-OHdG, TAS, TOS, NO, MPO, GSH and MDA levels, however, were not significantly different between groups (p>0.05).

**Conclusion:**

The findings of this study indicate that SRP plus gaseous ozone versus SRP alone does not correlate to a significant improvement in periodontal recovery.

## Introduction

The complicated interaction between the immune inflammatory response and periodontopathogenic bacteria usually results in chronic periodontitis (CP). In clinical terms, CP is characterized by periodontal pockets, gingival inflammation, and loss of attachment to and in the alveolar bone, which can eventually lead to tooth loss.^1^ A network of interacting molecular pathways involving proinflammatory mediators and reactive oxygen species (ROS) are involved in the progression of periodontal disease (PD).^2^ An increase in cytokine expression and immunological activity in gingival tissues occurs with this interaction between the host immune system and pathogenic bacteria, and proinflammatory mediators facilitate the immune response to periodontopathic bacteria. Local cytokine production, which develops in response to periodontal infection, may also influence the systemic environment. Tumor Necrosis Factor-α (TNFα) and Interleukin 6 (IL-6) are key cytokines for the initiation and maintenance of the systemic inflammation involved in the progression and severity of PD.^3^

Pathogenic mechanisms of the disease may involve antioxidant (AO) activity and oxygen-dependent ROS production. A study conducted by Chapple, et al.^4^ (1997) correlated PD with reduced salivary AO status, and increased oxidative damage in the oral cavity. Other studies have examined a possible relation between PD, reduced salivary AO status, and increased oxidative damage.^5^ ^,^ ^6^ An increase in levels of 8-hydroxy-2'-deoxyguanosine (8-OHdG), a biomarker of oxidative stress, has also been reported to be associated with periodontitis.^7^

Treatments for periodontitis focus on reducing pathogens embedded in the subgingival biofilm.^8^ Modalities range from oral hygiene instruction, non-surgical periodontal treatment (NSPT), and supra- and subgingival debridement, to periodontal surgery. NSPT may be applied by hand or via powered instrumentation. Studies conducted thus far indicate that NSPT reduces tooth loss risk, slows PD progression rate, and enhances gingival health, and NSPT is considered an indispensable therapy component against inflammatory PD.^9^ However, effectiveness of NSPT is limited in certain areas, such as difficulty to reach and deep pockets.^10^ The use of laser or ozone has been discussed as an alternative to this process, and recent reports include the use of ozone in dentistry as an alternative oral antiseptic component.^11^ According to recent studies, gaseous and aqueous forms of ozone have high antimicrobial power against oral pathogens and are impervious to resistance.^12^

Between 1880 and 1932, the ozone therapy method was widely used as an alternative medication in the United States, and many countries currently accept ozone therapy as a treatment modality.^13^ Ozone is an unstable matrix of three oxygen atoms (O_3_), and has been shown to be an impressive antiviral and antifungal agent.^14^ It has been used as a therapeutic agent for chronic wounds (e.g., trophic and ischemic ulcers, diabetic wounds, etc.) in several empirical studies.^15^ ^,^ ^16^ The use of ozone has been examined for efficacy against viral, fungal, and bacterial infections; ocular, ischemic, orthopedic, hematological, neurodegenerative, pulmonary, renal, and dermatological diseases; and age-dependent macular degeneration.^17^ ^,^ ^18^ Existing uses of both gaseous and aqueous forms of ozone in dentistry include inhibition of bacterial proliferation, enhancement of epithelial wound healing, enhancement of local oxygen supply, elimination of pathogen caries, root canal disinfection, and promotion of hemostasis.^19^ ^–^ ^21^ While laboratory studies suggest promise for ozone in the treatment of periodontal disease, clinical evidence is extremely limited. This study aims to evaluate the clinical and biochemical (oxidative stress and pro-inflammatory mediators) effects of the use of gaseous ozone in addition to scaling and root planning (SRP) in periodontal treatment.

## Materials and methods

### Study population

We utilized a randomized, parallel controlled, and double-blinded design in a full-mouth clinical trial with a follow up at one-month post-treatment. The Ethics Committee approved the study protocol in accordance with the Helsinki Declaration. This study was registered at ClinicalTrials.gov (NCT03444350). Written informed consent was obtained from all participants. Patients were selected from the Department of Periodontology patient pool. Forty patients with generalized to moderate CP were included in the study. Median age of patients was 42.4, within a deviation of ±6.7 years.

Sample-size estimation for this study was based on clinical attachment level (CAL), and it was determined that each group should be composed of 18 subjects. The test was calculated to provide 80% power, detecting differences of 0.5 mm within an SD of 0.6 mm. Two additional subjects were included per group (total 20) to compensate for possible dropouts during the study period.

To be included, patients were required to have generalized CP as agreed upon by consensus at the World Workshop in Periodontics in 1999;^22^ that is, three or more teeth having probing depth between 4 and 6 mm, in at least two quadrants, with radiographic signs of bone loss, and a minimum of 20 teeth. Patients who had received periodontal therapy within the last 12 months, had systemic disease which could affect periodontal treatment outcomes, or had taken systemic antibiotics within the last 6 months were excluded. Pregnant or breast-feeding patients were also excluded.

### Treatment protocol

SRP plus gaseous ozone [3 W, (n=20)] was applied to the experimental group. The control group (n=20) received SRP plus placebo. Patients were randomly distributed between the two groups. One patient in the treatment group and two in the control group left the study prior to completion.

**Figure 1:**
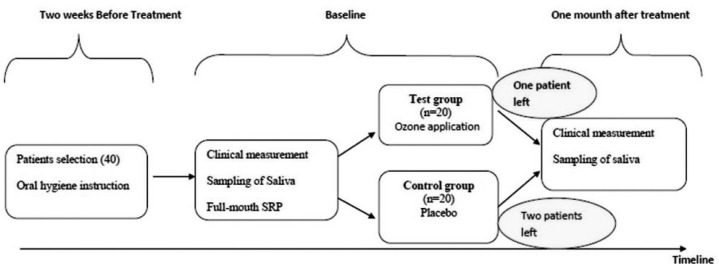
Research outline

**Figure 2:**
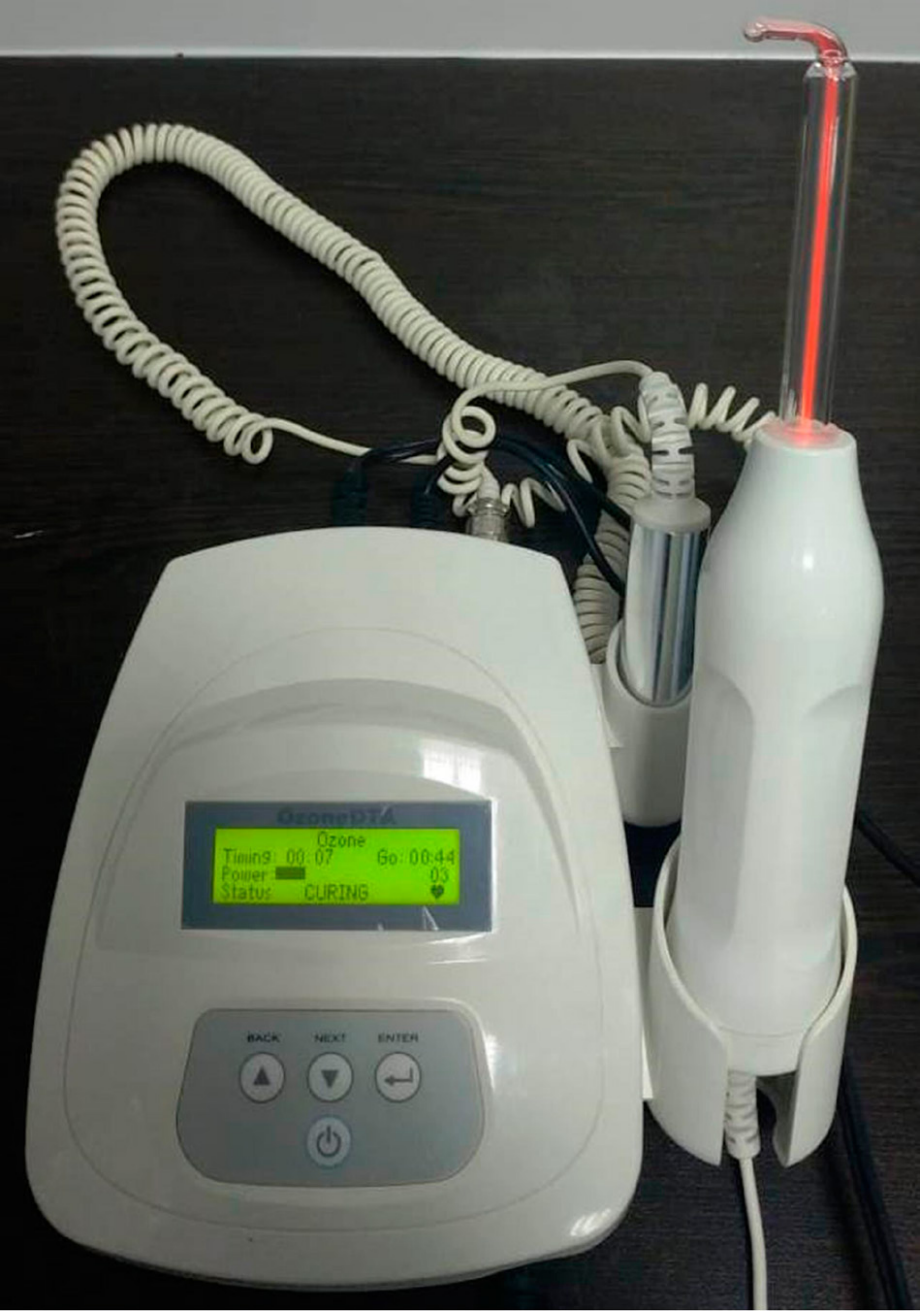
Ozone device used in the study

Participants were provided with detailed information on oral hygiene (including self-performed plaque control measures, and inter-dental cleaning maintenance with the help of flossing and toothbrushing) two weeks before treatment. Full-mouth supragingival and subgingival SRP was applied under local anesthesia, both by hand (Hu-Friedy, Chicago, IL, USA) and with ultrasonic instruments. Ozone was then applied to the periodontal pockets of patients in the experimental group in accordance with the manufacturer's protocol (Ozone DTA, Apoza Enterprise Co., New Taipei, Taiwan, R.O.C.) (Figure 2). Ozone application for each tooth was performed twice, on days 3 and 8, following periodontal treatment (Figure 1). Ozone was administered in the gingival sulcus of all teeth with a thin glass tip, moving in the apical-coronal direction in a sweeping motion, and applied to mesial, distal, buccal, and lingual surfaces in a one-minute period. The device was applied without ozone (placebo) in the control group.

### Randomization of the study

Forty patients were distributed randomly between the two treatment groups. One researcher maintained the randomization list until patients were found to be eligible for the study. The clinician who performed the therapy was not informed about the modality of the treatment until the beginning of the first session for each patient. Results were evaluated blindly before and after treatment. Clinical measurements for all patients were made by an examiner who was not involved in providing treatment during the study. Before the start of the study, the examiner was trained to perform adequate levels of accuracy and reproducibility in recording clinical parameters and indices.

### Clinical examination

One month after the second ozone application, plaque index (PI),^23^ gingival index (GI),^24^ probing depth, and CAL were recorded for each patient. A manual periodontal probe (PCP-12, Hu-Friedy, Chicago, IL, USA) was used for all clinical parameter measurements.

### Saliva collection and laboratory assessments

Saliva samples from all patients were taken via expectoration before treatment and one month after the second treatment application (ozone or placebo). Paraffin wax-stimulated, whole saliva samples were obtained from patients in a quiet room during clinical examination between 0900 and 1200, eight hours prior to food intake. Approximately 2 mL of whole saliva sample was collected in disposable tubes and immediately centrifuged at 1,000*g* for 10 minutes to remove cell debris. Supernatant was removed and stored in small aliquots at −80°C until analysis. Total antioxidant status (TAS), total oxidant status (TOS), nitric oxide (NO), 8-OHdG, myeloperoxidase (MPO), glutathione (GSH), malondialdehyde (MDA), and transforming growth factor-beta (TGF-β) levels were evaluated.

Photometric kits (Rel Assay Diagnostics Co., Gaziantep, Turkey) were used to determine TAS and TOS in the saliva samples according to manufacturer's directives. TOS results were given in terms of micromolar hydrogen peroxide equivalent *per* liter (μmol H_2_O_2_ equiv/L) and TAS results as mmol Trolox equiv/L.

Saliva 8-OHdG, TGF-β, and MPO levels were measured by enzyme-linked immunosorbent assay (ELISA) method. ELISA kits for TGF-β and MPO were purchased from Cayman Chemical Company (Cayman Chemical, Ann Arbor, HI, USA), and the ELISA kit for 8-OHdG was purchased from MyBioSource Inc. (MyBioSource, San Diego, CA, USA). 8-OHdG, MPO, and TGF-β levels in saliva samples were analyzed according to manufacturer's instructions. All ELISA analyses were performed on a Brio-SEAC semi-automatic ELISA machine (Radim Co., Calenzeno-Firenze, Italy).

MDA levels in saliva samples were examined using Uchiyama and Mihara method. In brief, spectrophotometric results measurements of the reaction between MDA and thiobarbituric acid were taken at 535 nm and stated as nmol/L.

Saliva NO levels were measured by Cortas' method, wherein spectrophotometric color density resulting from the reduction of nitrate to nitrite by cadmium is measured at 545 nm and expressed in μmol/L.

Saliva GSH values were calculated by the method developed by Fairbanks and Clee; that is, spectrophotometric measurement of the color resulting from the reaction between sulfhydryl groups and Elman's reagent at 410 nm. Values are expressed as μmol/L.

### Statistical analyses

Data was analyzed using SPSS software version 17.0 for Windows. Means and standard deviations were calculated, and *p* values <0.05 were considered statistically significant. Shapiro-Wilk tests were computed for each variable to assess whether the variables were distributed normally. Student's *t*-tests were used to determine the significance of differences in clinical parameters between groups. Differences in laboratory findings between groups were analyzed by the Mann-Whitney U-test. Wilcoxon tests were administrated to explore differences within groups.

## Results

Socio-demographic characteristics of the study participants are shown in Table 1. No significant differences among participants were found in terms of gender, mean age, smoking rate and educational status.

**Table 1:** Characteristics of the subjects

	Test Group n=19	Control Group n=18	p
Female/Male (n)	8/11	8/10	NS
Mean age (years, mean±SD)	44.7±5.1	45.8±5.6	NS
Smoking (n) (yes)	10	9	NS
Education (years, mean±SD)	10.6±2.7	9.9±3.0	NS

NS=not significant.

Baseline clinical measurements were similar for both groups. Postoperative improvement in all clinical parameters was noted for both groups (*p*<0.05). After treatment, changes in the PI, GI, probing depth, and CAL were also similar for both groups (*p*>0.05) (Table 2).

**Table 2:** The compared inter- and intragroup clinical findings at baseline and one month after treatment (mean±SD)

	Baseline	One-month after treatment	Change	p
PI				
Test Group	2.5±0.6	0.6±0.2	1.9	0.0003
Control Group	2.4±0.6	0.6±0.2	1.8	0.0003
p	N.S.		NS	
GI				
Test Group	2.3±0.7	0.8±0.2	1.4	0.0006
Control Group	2.1±0.6	0.9±0.2	1.2	0.0009
p	NS		NS	
Probing depth				
Test Group	3.8±0.8	3.0±0.6	0.8	0.008
Control Group	3.6±0.8	3.0±0.8	0.6	0.009
p	NS		NS	
CAL				
Test Group	4.4±1.1	4.0±0.7	0.4	0.03
Control Group	4.1±0.8	3.8±0.8	0.3	0.03
p	NS		NS	

PI=plaque index; GI=gingival index; CAL=clinical attachment level

NS=not significant

p<0.05; statistically significant

Baseline laboratory findings were similar for both groups (*p*>0.05). The increase of MPO and TAS was observed in both groups after treatment (*p*<0.05). However, the increase of TGF-β levels was significantly greater in the test group compared with controls (*p*<0.05). Levels of 8-OHdG decreased in both groups after treatment (*p*<0.05) (Table 3).

**Table 3:** The compared inter- and intra the groups of laboratory findings at baseline and at one month after treatment (mean±SD)

	Baseline	One-month after treatment	Change	p
8 OHdG				
Test Group	1331±451	1045±309	-286	0.008
Control Group	1626±502	1324±327	-302	0.008
p	NS		**0.047**	
GSH				
Test Group	30.9±14.7	35.1±17.2	4.2	NS
Control Group	29.6±14.9	34.2±16.5	4.6	NS
p			NS	
MDA				
Test Group	23.4±8.1	18.0±7.3	-5.4	NS
Control Group	20.6±7.9	16.9±7.9	-3.7	NS
p	NS		**N.S.**	
MPO				
Test Group	752±294	854±306	102	0.042
Control Group	651±304	761±354	100	0.038
p	NS		NS	
TAS				
Test Group	4.33±1.1	4.72±1.2	0.39	0.038
Control Group	4.01±1.0	4.51±1.0	0.5	0.024
P	NS		NS	
TOS				
Test Group	16.2±5.5	15.5±5.6	0.7	NS
Control Group	17.3±6.1	15.3±6.0	2	NS
P			NS	
TGF- β				
Test Group	42.7±11.6	48.4±11.1	5.7	0.031
Control Group	39.5±10.2	39.6±10.4	0.1	NS
P	NS		**0.026**	
NO				
Test Group	24.5±9.8	20.9±6.1	3.6	NS
Control Group	23.3±8.6	20.0±6.6	3.3	NS
p	NS		**N.S.**	

8-OHdG=8-hydroxy-2'-deoxyguanosine; GSH=glutathione; MDA=malondialdehyde; MPO=myeloperoxidase; TAS=total antioxidant status; TOS=total oxidant status; TGF-β=transforming growth factor beta; NO=nitric oxide

NS=not significant

p<0.05; statistically significant

Changes in 8-OHdG, GSH, MDA, MPO, TAS, TOS, and NO levels were similar between groups (*p*>0.05). Statistically significant differences between the ozone and control groups were limited to TGF-β levels following treatment (*p*<0.05) (Table 3).

## Discussion

This is the first study to evaluate both clinical and biochemical *in vivo* effects of gaseous ozone on oxidative stress (TAS and TOS) and TGF-β, a marker for periodontal recovery. Ozone was applied in addition to noninvasive periodontal treatment. Levels of MDA, a lipid peroxidation end product; GSH, an AO molecule; 8-OHdG, a marker of purin oxidation product; and NO, an oxidant agent were assessed. Results showed that the application of gaseous ozone was not associated with significant changes in periodontal recovery. Primary goals for the treatment of PD are the recycling of attachment loss, the decrease in pocket depth, and the elimination of inflammation.^25^ In order to reach these goals, the first treatment option for patients whose pockets are not deep is non-surgical.^26^ However, the success of non-surgical treatment with hand tools and cavitron is limited where pockets are difficult to reach.^10^ In addition, the effectiveness of non-surgical treatment in tissue regeneration, a primary therapeutic target, is less than ideal. As such, changing the host immune response to oral pathogens and increasing the efficiency of treatment methods are desired markers for clinical success. Recently, ozone treatment has been hypothesized to increase tissue healing and periodontal recovery by decreasing oxidative stress and antibacterial characteristics. In this study, we aimed to assess the effectiveness of gaseous ozone on periodontal tissue recovery by examining clinical and laboratory findings.

The use of ozone in periodontal therapy is based on its antimicrobial, immunostimulating, anti-hypoxic, and biosynthetic properties.^27^ Ozone is theorized to contribute to periodontal healing by eliminating pathogens, activating the immune system, and stimulating the humoral antioxidant system;^27^ however, clinical evidence thereto is limited. Most studies on this modality are carried out *in vitro* and few clinical trials have been done. We examined the clinical effects of ozone use in periodontal treatment using a randomized, double-blinded study design.

Nagayoshi, et al.^28^ (2004) have reported that ozonated water was effective in killing both gram-positive and gram-negative oral microorganisms. They also reported that gram-negative anaerobes were more sensitive to ozonated water than gram-positive anaerobes. Their study found that ozonated water could inhibit the accumulation of experimental dental plaque *in vitro* and reported that ozonated water had strong activity against bacteria found in plaque biofilms.^27^ Muller, et al.^29^ (2007) compared the efficiency of gaseous ozone versus photodynamic therapy on oral biofilms, however, they found that the use of ozone could not eliminate or reduce biofilm microbial populations. Huth, et al.^11^ (2007) studied the influence of ozone on the host immune response and concluded that NF-κB activity in periodontal ligament tissue from the root surfaces of periodontally damaged teeth was inhibited following incubation with ozonated media. In this context, Huth's team contended that ozone application could induce anti-inflammatory effects.

Many recent studies have investigated the effects of ozonated water and SRP plus ozone applications. Ramzy, et al.^30^ (2005) did a study on 22 aggressive periodontitis patients wherein ozonated water was used to irrigate their periodontal pockets. Significant improvements in PI, GI, probing depth, and bacterial count were observed; however, they also reported statistically significant decreases in bacterial counts in areas that received ionized water treatment. Kshitish and Laxman^31^ (2010), in a study with generalized CP patients, irrigated one half of the mouth with ozonated water and the other half with 0.2% chlorhexidine andreported better results with ozonated water compared to chlorhexidine irrigation in PI, GI, and bleeding indices. In a study conducted on CP patients by Yilmaz, et al.^32^ (2013), the effects of the Er:YAG laser and topical gaseous ozone application (as adjuncts to initial periodontal therapy) were clinically and microbiologically investigated; however, their results showed attachment gain and probing depth reduction at a statistically significant level in favor of the laser group, although intergroup comparisons of microbiological parameters showed no significant differences. Habashneh, et al.^33^ (2015), in another study on CP patients, reported no significant differences between SRP + irrigation with ozonated water versus SRP + irrigation with distilled water in terms of PI, GI, bacterial proliferation, probing depth, gingival recession, and CAL and serum CRP levels. Evidence regarding ozone application on clinical recovery, therefore, has been difficult to pin down.

Recent clinical studies have demonstrated that gaseous and aqueous forms of ozone have antimicrobial effects against oral pathogens linked with caries and endodontic infections. There have also been reports that ozone therapy decreases the growth of *Aggregatibacter actinomycetemcomitans, Porphyromonas gingivalis*, and *Tannerella forsythia* at statistically significant levels.^34^ ^,^ ^35^ Some studies, even in aggressive periodontitis patients, assaying SRP plus ozone have also demonstrated significant improvements in GI, pocket probing depths, PI, and bacterial counts.^30^ ^,^ ^31^ Hauser-Gerspach, et al.^36^ (2012) examined the effects on bacteria adhered to titanium implant surface of gaseous ozone and reported decrease in bacterial counts in the surfaces that received ozone treatment.

Despite results in the literature suggesting ozone might be influential in the elimination of periodontopathogenic bacteria, data regarding clinical recovery are not consistent with these findings. However, ozone application has been associated with increased bone and epithelial recovery.^37^ ^,^ ^38^ Based on these data, it might be expected that ozone could exert a positive influence on clinical recovery from PD. Our clinical results, however, indicated that ozone treatment was not correlated with additional benefits in clinical periodontal recovery within a one-month period.

Ozone has several positive effects on cellular and humoral immune system components, in stimulation of the proliferation of immunocompetent cells and synthesis of immunoglobulins.^39^ It has been well-established that ozone activates macrophages and enhances the sensitivity of microorganisms to phagocytosis. Immune cells of the body produce certain cytokines as a response to the activation caused by ozone. Ozone triggers the synthesis of various biologically active substances that reduce inflammation and promote wound healing, such as interleukins, leukotrienes and prostaglandins. Oxidant and AO molecules are also known to play important roles in the immune response to PD.^2^ For this purpose, we evaluated the effects of ozone treatment on oxidants and AOs by assaying levels of TOS, NO, 8-OHdG, MDA, GSH, and TAS in saliva to investigate the ozone effects on the immune response in PD. According to our findings, although increase in AO levels and decrease in oxidant levels were more pronounced in the ozone-treated group, no statistically significant differences were observed for any specific clinical parameters. These findings suggest that the stimulatory effects of ozone administration on periodontal tissues may be limited.

In addition, TGF-β levels were evaluated as a biochemical marker for tissue recovery. Previous studies have reported that an increase in TGF-β levels was associated with a decrease in periodontal inflammation, leading to improved outcomes.^40^ We did observe that TGF-β levels increased significantly in the ozone-treated group, supporting the claim that gaseous ozone treatment increases the regeneration activity of periodontal tissues. The difference in TGF-β levels could indicate that ozone therapy can contribute to periodontal treatment; however, we found that clinical recovery and changes to biochemical parameters were similar for both groups, confounding this result. The ozone application effect on whole oxidant and AO levels may be limited due to multiple factors involved in immune response and recovery.

Follow-up was limited to one month after second treatment application, which did not permit the evaluation of long-term periodontal outcomes. Although this period was beneficial in terms of eliminating the differences in hygiene practices among the patients and evaluating the short-term host responses, further studies on the long-term effects of ozone treatment on PD are needed.

## Conclusion

SRP plus ozone application in periodontal pockets demonstrated similar effects as SRP alone in clinical recovery indicators and changes to biochemical markers. However, further research on the differential effects of ozone parameters, such as dosage, duration, and usage frequency in periodontal healing is needed.
